# Irreducible inguinal hernia with appendices epiploicae in the sac

**DOI:** 10.4103/0972-9941.43094

**Published:** 2008

**Authors:** Mayank Jain, Shashi Khanna, Bimalendu Sen, Om Tantia

**Affiliations:** Department of Minimal Access Surgery, ILS Multispeciality Clinic, DD-6, Sector – I, Salt Lake City, Kolkata – 700 064, India

**Keywords:** Appendices epiploicae, Inguinal Hernia, Trans-abdominal pre peritoneal

## Abstract

Inguinal hernia has a nature to surprise surgeons with its unexpected contents. Appendix epiploicae alone in the hernial sac is a rare entity and that too if hypertrophied and presenting as irreducible hernia is still more uncommon. We report a 52-year-old male with complains of irreducible inguinal mass with little pain on Left side for seven days. A diagnosis of irreducible inguinal hernia was made and the patient was treated laparoscopically by Trans-Abdominal Pre-Peritoneal Mesh Hernioplasty (TAPP). As a surprise, content of the hernial sac was enlarged / hypertrophied appendix epiploicae of sigmoid colon with appendigitis. Patient also had and incidental hernia on the other side, which was repaired in the same sitting. Postoperative recovery of the patient was excellent.

## INTRODUCTION

Nearly all abdominal organs, even the stomach have been found within the inguinal hernia sac.[1] So even after vast experience it is not uncommon for a surgeon to see something unexpected. Epiploic appendices were first described by Vesalius in 1543.[2] Epiploic appendix in the sac of inguinal hernia with appendagitis is very rare and not many cases have been reported in the literature. The medline search shows case reported from Turkey in 1989 and Russia in 2005.[3,4] We report a new case where hypertrophied epiploic appendix was the sole content of irreducible inguinal hernia sac.

Hypertrophied appendix epiploicae was reduced and excised. An incidental right direct hernia was also detected and bilateral tension free Trans-abdominal pre peritoneal (TAPP) mesh hernioplasty was performed.

## CASE REPORT

A 52-year-old male presented with complains of pain and swelling in the Lt. Groin which was irreducible since last seven days. There was history of (h/o) swelling since last three years but it had been painless and reducible all over those years. There was no history of chronic cough / constipation / micturation problems. There was no history suggestive of bowel obstruction. He did not suffer from any significant medical/surgical disease in the past. On examination there was a painless mass of about 3 cm size in the left inguinal region. There was no cough impulse. No other abnormality was detected. Patient was diagnosed as irreducible Left inguinal hernia. Routine investigations were within the normal limits. The ultrasound showed inguinal wall defect with some content. An informed consent was obtained and he was planned for Laparoscopic Left Inguinal Hernioplasty (TAPP) with possibility of conversion to open.

**Figure 1:**
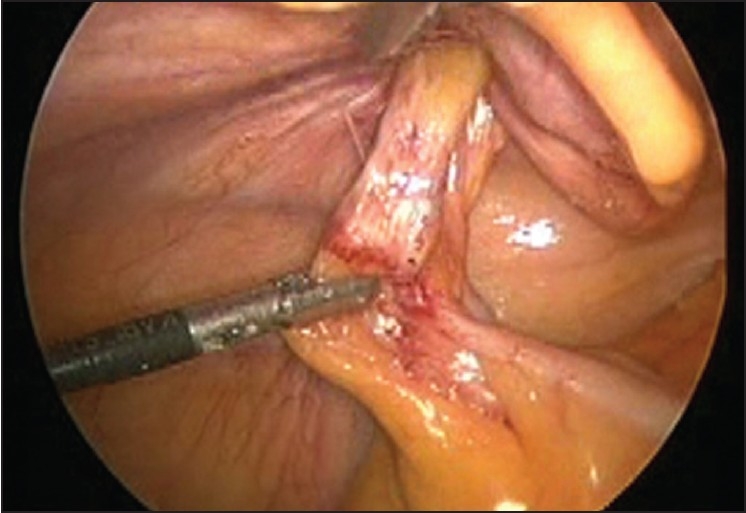
Irreducible left inguinal hernia (direct)

**Figure 2:**
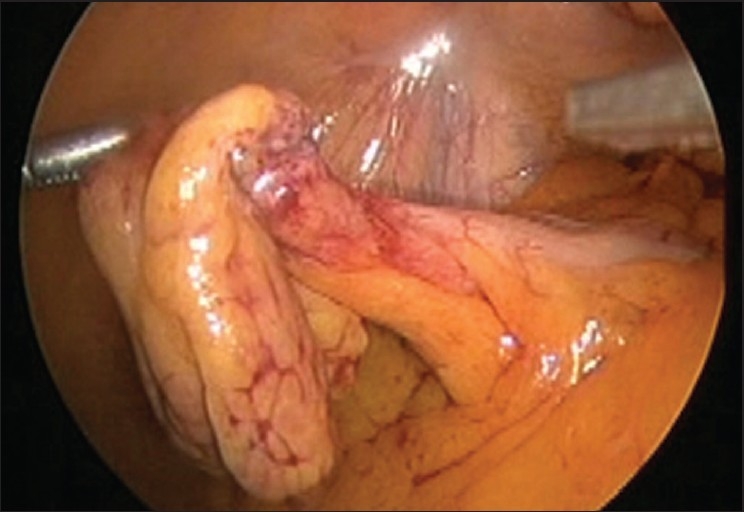
Hypertrophied inflamed appendicic epiploicae

Peri-operatively, there was a direct hernia on the left side. The content of the hernia was appendices epiploicae of sigmoid colon [Figure 1] and was adhered to the sac but could be reduced. This epiploic appendix was hypertrophied and inflamed [Figure 2] and so was resected with the help of ultrasonic shears. The hernia was repaired by TAPP method in the standard manner with the use of a polypropylene mesh (12 × 15 cm).

Right side direct inguinal hernia was also seen on internal view as incidental finding and was repaired by same technique using separate similar size Prolene Mesh in the same sitting. Post-operative period was uneventful. Patient had smooth recovery, and was started on full oral diet since next morning and discharged on day 2. On Routine follow-up at Day 7 and Day 30 there was no complaint.

## DISCUSSION

Appendices epiploicae are fat-containing peritoneal out pouchings arising from the serosal surface of the colon. They can be found at any point between the caecum and rectosigmoid colon. Their length may vary between 0.5 and 5.0 cm.[5] Appendix epiploicae as a content of the inguinal hernial sac is a rare phenomenon and very few cases have been reported by now.[3,4] Further, hypertrophied and inflamed epiploicae is also rare with only a few hundred cases reported by now.

Epiploic appendicitis usually presents with abdominal pain, nausea, vomiting, anorexia and low grade fever.[6] It is a self-limiting condition and conservative treatment has been advocated by some centres.[7] A computerised tomographic scan (CT Scan) is important in formulating the principles of treatment.[8,9] Inflammation of the appendix epiploicae may be primary or secondary to various factors. Incarceration within the hernial sac is however a rare cause for secondary torsion. Also, an already inflamed appendix may enter the hernial sac and incarcerate.

Although epiploic appendicitis is a self-limiting disease and may improve conservatively with analgesics and nonsteroidal anti-inflammatory agents over a few days, but the surgery is the right option for the cases presenting like this. This is because the possibility of incarcerated hernia cannot be ruled out. Also it has been advised that any inguinal hernia should be repaired on elective basis.[10] It is for these reasons that this case required surgical management. Since the laparoscopic technique was used, we could identify the incidental hernia on the opposite side (right side) which was also repaired in the same sitting.

To conclude, it is nearly impossible to preoperatively predict epiploic appendix in the hernial sac. However this may not be a real problem since the treatment does not differ and surgical management is the right choice. Also laparoscopic technique can be used for such irreducible hernias which have an advantage of detecting incidental hernia on the other side.
